# The prevalence of soil transmitted helminths and its influential factors in Shandong Province, China: an analysis of surveillance data from 2016 to 2020

**DOI:** 10.1186/s40249-023-01100-4

**Published:** 2023-05-22

**Authors:** Yan Xu, Yongbin Wang, Longjiang Wang, Xiangli Kong, Ge Yan, Yuejin Li, Cancan Bu, Benguang Zhang

**Affiliations:** Shandong Institute of Parasitic Diseases, Shandong First Medical University & Shandong Academy of Medical Sciences, No.11 Taibai Middle Road, Rencheng District, Jining City, 272033 Shandong Province People’s Republic of China

**Keywords:** Soil-transmitted helminths, Surveillance, Prevalence, Influential factor, Shandong Province, China

## Abstract

**Background:**

Soil-transmitted helminths (STHs) were previously endemic in Shandong Province, China. This study aimed to analyze the STHs prevalence trend and the natural, social, and human cognitive and behavioural factors influencing the discrepancies between high and low infection levels from 2016 to 2020 in Shandong Province in eastern China.

**Methods:**

STHs surveillance data of Shandong Province from 2016 to 2020 were obtained from China Information Management System for Prevention and Control of Parasitic Diseases. STHs infections were detected by modified Kato–Katz method. Comprehensive information on the natural and social factors, STHs-related knowledge and behaviours were collected through questionnaire surveys. Retrospective spatial scan analysis was performed using SaTScan v10.1 to evaluate any identified spatial clusters of STHs infection for statistical significance and Bayes discriminant analysis was used to discriminate the high or low infection groups of the villages.

**Results:**

In total, 72,160 participants were involved in our survey from 2016 to 2020. The overall STHs prevalence rate was 1.13%, with the eastern region of Shandong Province having the highest rate (2.02%). The predominant species was *T. trichiura*, with the prevalence rate of 0.99% and the ≥ 70-year age group possessed the highest rate of 2.21%. The STHs prevalence rate showed an annual linear downward trend from 2016 to 2020 ($\chi_{{{\text{trend}}}}^{2}$ = 127.600, *P* < 0.001). Respondents aged ≥ 60 years had the lowest awareness level of STHs-related prevention knowledge (all *P* < 0.05), and were the most likely to adopt the practice of fertilizing with fresh stool (*χ*^*2*^ = 28.354, *P* < 0.001). Furthermore, the southern region demonstrated the highest temperature and rainfall level and the lowest GNP and annual net income per capita (all *P* < 0.05).

**Conclusions:**

There is a remarkable declining in STHs prevalence in Shandong Province from 2016 to 2020. However, the prevalence rates of STHs especially *T. trichiura* in the southern and eastern regions were still high, and the elderly were more susceptible to be infected with STHs owning to their low awareness level of STHS-related prevention knowledge and high adoption rate of dangerous production and living behaviours. Integrated approaches of health education, environment improvement and behaviour change should be strengthened to obtain a further reduction of STHs prevalence in China.

**Supplementary Information:**

The online version contains supplementary material available at 10.1186/s40249-023-01100-4.

## Background

Soil-transmitted helminths (STHs) are a group of parasitic nematodes that cause human infection through contact with parasite eggs or infective larval stages [1, 2]. The primary STHs species are the roundworm (*Ascaris lumbricoides*), whipworm (*Trichuris trichiura*), and hookworm (*Necator americanus* and *Ancylostoma duodenale*) [3, 4]. STHs are one of the causes of physical and intellectual growth delays worldwide [5–7], especially in children, STHs can lead to malnutrition, stunted growth, intellectual delays, and cognitive and educational deficits [8–10]. Over 1.5 billion people, or 24% of the world’s population, are infected with STHs [11]. STHs infections are widely distributed in tropical and subtropical areas such as sub-Saharan Africa, the Americas, and East Asia [12–17]. Additionally, STHs infections are recognized as neglected tropical diseases (NTDs), with a disease burden of 1.9 million disability-adjusted life years (DALYs) in 2019 in the world [18, 19].

STHs infection was a serious threat to public health in China. However, rapid socioeconomic development and large-scale helminth control activities resulted in a significant decline in STHs prevalence in recent years [16, 20, 21]. Notably, national survey on distribution of human parasites, national survey on the status of human key parasitic diseases, and national survey on current status of major human parasitic diseases implemented in 1988–1992, 2001–2004, and 2014–2016 revealed STHs prevalence rates of 53.58%, 19.56%, and 4.49%, respectively. [22–24]. China’s progress has made it the “most feasible” country in which STHs transmission interruption can be achieved [25]. STHs epidemiology in China was characterized by regional distribution in the 2014–2016 national survey [24]. Thus, the feasibility of transmission interruption should be further assessed at the provincial level [16].

Shandong Province is a major endemic region of STHs in China. Specific geographical environments and climatic conditions are suitable for STHs survival and reproduction. In the 1988–1992 national survey, the prevalence rates of *A. lumbricoides*, hookworm, and *T. trichiura* were 38.77%, 4.00%, and 13.84%, respectively, with *A. lumbricoides* as the dominant species [26]. The natural environments and living conditions in rural areas of Shandong Province underwent substantial changes with the continuous development of China’s social economy. The prevalence of *A. lumbricoides*, hookworm, and *T. trichiura* decreased to 6.37%, 0.50%, and 9.89%, respectively, in the 2001–2004 national survey and 0.16%, 0.02%, and 0.58%, respectively, in the 2014–2016 national survey. *T. trichiura* became the dominant species*,* and the STHs prevalence rate of the province was in the top five in China [23, 27]. The *A. lumbricoides*, hookworm, and *T. trichiura* prevalence rates in 2014–2016 had reduced by 99.59%, 99.50%, and 95.81%, respectively, compared to those observed in the 1988–1992 national survey.

Since 2016, fourteen counties were selected as sites for conducting STHs surveillance annually in Shandong Province. This retrospective study used surveillance data from 2016 to 2020 of Shandong Province to describe the epidemiological characteristics of STHs, to capture the STHs prevalence trend and to identify potential factors influencing the discrepancies between high and low infection levels. These findings might contribute to developing and improving STHs prevention and control measures and help in national STHs transmission interruption.

## Methods

### Surveillance site and participants

Shandong Province is located in the east of China downstream from the Yellow River and lies 34° 22.9′–38° 24.01′ North and 114° 47.5′–122° 42.3′ East. The province is dominated by mountainous and hilly terrain. The province has a warm, temperate monsoon climate characterized by a mild climate, concentrated rainfall, and four distinct seasons. The province is comprised of 16 prefecture-level cities and 136 counties. From 2016 to 2020, fourteen counties were selected as STHs surveillance sites annually in Shandong Province. Five different villages were randomly sampled from five different townships of each surveillance site, and no fewer than 200 people over 3 years of age were investigated from each village. And the surveillance sites were divided into four regions: central, eastern, southern, and northern region according to their geographical location in Shandong Province.

### STHs infection examination

Stool samples (> 30 g) were collected from each participant and examined for parasite eggs using the modified Kato–Katz method [28] (two smears for each stool sample). STHs infection was defined as one or more eggs of *A. lumbricoides*, hookworm, or *T. trichiura* in either of the two smears. The infection intensities of the three helminth species were categorized as light, moderate, and heavy, according to the World Health Organization (WHO) guidelines [29]. The basic information and examination results of all participants were entered into China Information Management System of Parasitic Diseases Prevention and Control.

### Questionnaire survey

Participants were selected randomly at a sampling proportion of 2%, and questionnaire survey was conducted using the “Health Knowledge and Behaviour Questionnaire”. Information regarding natural and social factors, such as the geographical environment, temperature, humidity, rainfall, economic level, and sanitary conditions of five villages in each surveillance site was collected according to the “Questionnaire on Basic Information of STHs Surveillance Site”. Both questionnaires were compiled and authorized by the National Institute of Parasitic Diseases at Chinese Center for Disease Control and Prevention (China CDC).

### Quality control

Four measures were implemented for quality control: (1) Professionals from the county CDC were divided into different groups, namely sample collection, questionnaire survey, smear preparation, microscope examination, and data reporting groups. Each group was trained by experts from the Shandong Institute of Parasitic Diseases to ensure they had mastered the surveillance techniques; (2) Professionals from provincial or municipal agencies were dispatched to guide the on-site and laboratory work throughout the surveillance process; (3) A provincial agency reviewed all positive samples and 5% of the negative samples to check the accuracy of the examination results, and the coincidence rate was required to reach 100%; otherwise, all samples needed to be re-examined; (4) Provincial professionals examined the integrity and logic of the data, and verified and corrected the problems in the surveillance data reported by the county CDC.

### Data analysis

Categorical variables were expressed as percentages, and normally distributed quantitative variables were presented as means and standard deviations. The Chi-square test was used to compare rates and for the trend analysis, and a one-way analysis of variance was used to compare the differences in the natural and social factors. The Bonferroni-corrected post-hoc test was conducted to adjust the significant level for multiple comparisons. All statistical analysis were performed using SPSS 26.0 (IBM-SPSS Inc., Chicago, IL, USA). Retrospective spatial scan analysis was performed to evaluate any identified spatial clusters of STHs infection for statistical significance using SaTScan v10.1 (Management Information Services, Maryland, USA). The Bernoulli probability model was applied using a circular window and with a maximum spatial cluster size of 50% of the population at risk for high-rate clusters. The cluster with the maximum log likelihood ratio (*LLR*) value was defined as primary cluster, while other clusters with statistically significant *LLR*s were defined as secondary clusters. The surveillance village was classified into high infection group when STHs prevalence > 3%, otherwise were classified into low infection group. Bayes discriminant analysis was used to discriminate the villages’ high or low infection groups using known natural and social factors, and the village was classified into the group which with the larger classification function value. *P* < 0.05 indicated statistically significant.

### Ethical approval and informed consent

The ethical review committee of the National Institute of Parasitic Diseases at Chinese CDC approved the surveillance (Approval No: 2021006). The objectives of the study were orally explained, and all participants were informed and written consent forms were obtained with either the signature of the participants or their guardian.

## Results

### STHs prevalence

From 2016 to 2020, a total of 72,160 participants were involved in this study, including 34,525 males and 37,635 females (Additional file 1: Fig. S1). The result showed that the total prevalence rate of STHs was 1.13% (819/72,160), among which the prevalence rate of *A. lumbricoides* was 0.13% (92/72,160), hookworm was 0.03% (23/72,160) and *T. trichiura* was 0.99% (716/72,160). According to the intensity of infection, *A. lumbricoides*, hookworm, and *T. trichiura* were predominantly mildly infected, with proportion of 95.65%, 95.65% and 96.09%, respectively. In addition, 1.47% (12/819) of STHs infection were mixed infections, with predominant mixed infections of *A. lumbricoides* and *T. trichiura* (91.67%, 11/12), and the rest were mixed with *T. trichiura* and hookworms (8.33%, 1/12) (Table 1).

**Table 1:** Prevalence and infection intensity of STHs species in Shandong Province

Helminth	Total infection		Light infection			Moderate infection			Heavy infection		
	*n*	Prevalence rate (%)	Standards (EPG)	*n*	Proportion (%)	Standards (EPG)	*n*	Proportion (%)	Standards (EPG)	*n*	Proportion (%)
*Ascaris lumbricoides*	92^a^	0.13	< 5000	88	95.65	5000–49,999	4	4.35	≥ 50,000	0	0
Hookworm	23^b^	0.03	< 2000	22	95.65	2000–3999	0	0	≥ 4000	1	4.35
*Trichuris trichiura*	716^c^	0.99	< 1000	688	96.09	1000–9999	26	3.63	≥ 10,000	2	0.28

*STHs* soil-transmitted helminths

^a^Among 92 *A. lumbricoides* infected cases, 11 cases were mixed infection with *T. trichiura*

^b^Among 23 hookworm infected cases, 1 case was mixed infection with *T. trichiura*

^c^Among 716 T*. trichiura* infected cases, 11 cases were mixed infection with *A. lumbricoides* and 1 case was mixed infection with hookworm

### Epidemiological characteristics

Data analysis showed that 62.90% of the surveillance counties had participants with STHs infection; among them, Yinan of Linyi City had the highest prevalence rate of 10.74% (109/1015), followed by Haiyang of Yantai City, Rongcheng of Weihai City and Lanshan of Rizhao City, with prevalence rates of 10.18% (102/1002), 8.76% (88/1004), 8.06% (82/1018), respectively. While the prevalence rates of other surveillance counties were below 5%, and the average rate was 0.98%, with the highest prevalence rate was 4.46% (45/1010) in Junan of Linyi City and the lowest prevalence rate was 0.09% (1/1089) in Yucheng of Dezhou City (Table 2).

**Table 2:** STHs prevalence of surveillance counties in Shandong Province

Infection rate (%)	Counties	No. surveillance	No. infection	Average prevalence rate (%)
> 10	Yinan, Haiyang	2017	211	10.46
5.00–9.99	Rongcheng, Lanshan	2022	170	8.41
1.00–4.99	Junan, Juxian, Wendeng, Linshu, Hedong, Donggang, Huancui, Yishui, Lanshan, Daiyue, Boshan, Tancheng	16,461	327	1.99
0.01–0.99	Linqu, Wulian, Mudan, Gangcheng, Zhifu, Yiyuan, Guangrao, Changdao, Liangshan, Yuncheng, Yutai, Penglai, Xuecheng, Jimo, Laiyang, Zhaoyuan, Laixi, Lanling, Laiwu, Wenshang, Yicheng, Juancheng, Yucheng	28,076	111	0.40
0	Anqiu, Caoxian, Changle, Changyi, Dongping, Dongying, Feicheng, Gaoqing, Jiaxiang, Jinxiang, Juye, Fushan, Laizhou, Lijin, Linyi, Linzi, Pingyin, Qingzhou, Shanghe, Sishui, Zhangdian, Zhangqiu, Changqing	23,584	0	0.00

According to geographical location, the prevalence rate in the central, eastern, southern, and northern regions of Shandong Province was 0.25% (48/19,456), 2.02% (268/13,264), 1.45% (496/34,144) and 0.13% (7/5296), respectively, and the difference was statistically significant (*χ*^2^ = 307.675, *P* < 0.001). The two-pair comparison showed that the difference between the central and northern region was not statistically significant (*χ*^2^ = 2.463, *P* = 0.117), while the prevalence rate of eastern region was higher than other three regions and the prevalence rate of southern region was higher than central and northern region (all *P* < 0.001) (Additional file 1: Table S1).

The spatial scan analysis revealed one primary cluster and three secondary clusters. The primary cluster (a) was located at 35° 17′ N and 119° 11′ E with a radius of 76.35 km, covering eight counties of southern region of Shandong Province. This cluster had 6,152,451 exposed persons, with a risk of STHs infection 6.08 times of the surrounding areas (*P* < 0.001). The secondary cluster 1 (b) and secondary cluster 3 (d) demonstrated some overlap with the primary cluster and were also located in the southern region. Secondary cluster 2 (c) was located at 37° 22′ N and 122° 6′ E with a radius of 106.39 km, covering six counties of eastern region. This cluster included 4,170,756 exposed persons, and the *RR* of this cluster was 4.73 (*P* < 0.001) (Table 3).

**Table 3:** Spatial clusters of STHs prevalence in Shandong Province

Clusters	Covering counties	Radius (km)	Number of cases	Expected cases	*LLR*	*RR*	*P*-value
Primary cluster (a)	Lanshan, Donggang, Junan, Wulian, Juxian, Hedong, Linshu, Yinan	76.35	360	93.58	279.91	6.08	< 0.001
Secondary cluster 1 (b)	Juxian, Wulian, Yishui, Yinan, Donggang, Lanshan, Junan	51.05	319	81.71	244.74	5.76	< 0.001
Secondary cluster 2 (c)	Huancui, Wendeng, Rongcheng, Zhifu, Fushan, Haiyang	106.39	250	69.55	166.08	4.73	< 0.001
Secondary cluster 3 (d)	Hedong, Lanshan, Linshu	30.55	122	81.32	10.08	1.59	0.001

*STHs* Soil-transmitted helminths, *LLR* Log-likelihood ratio, *RR* Relative risk

**Fig. 1:**
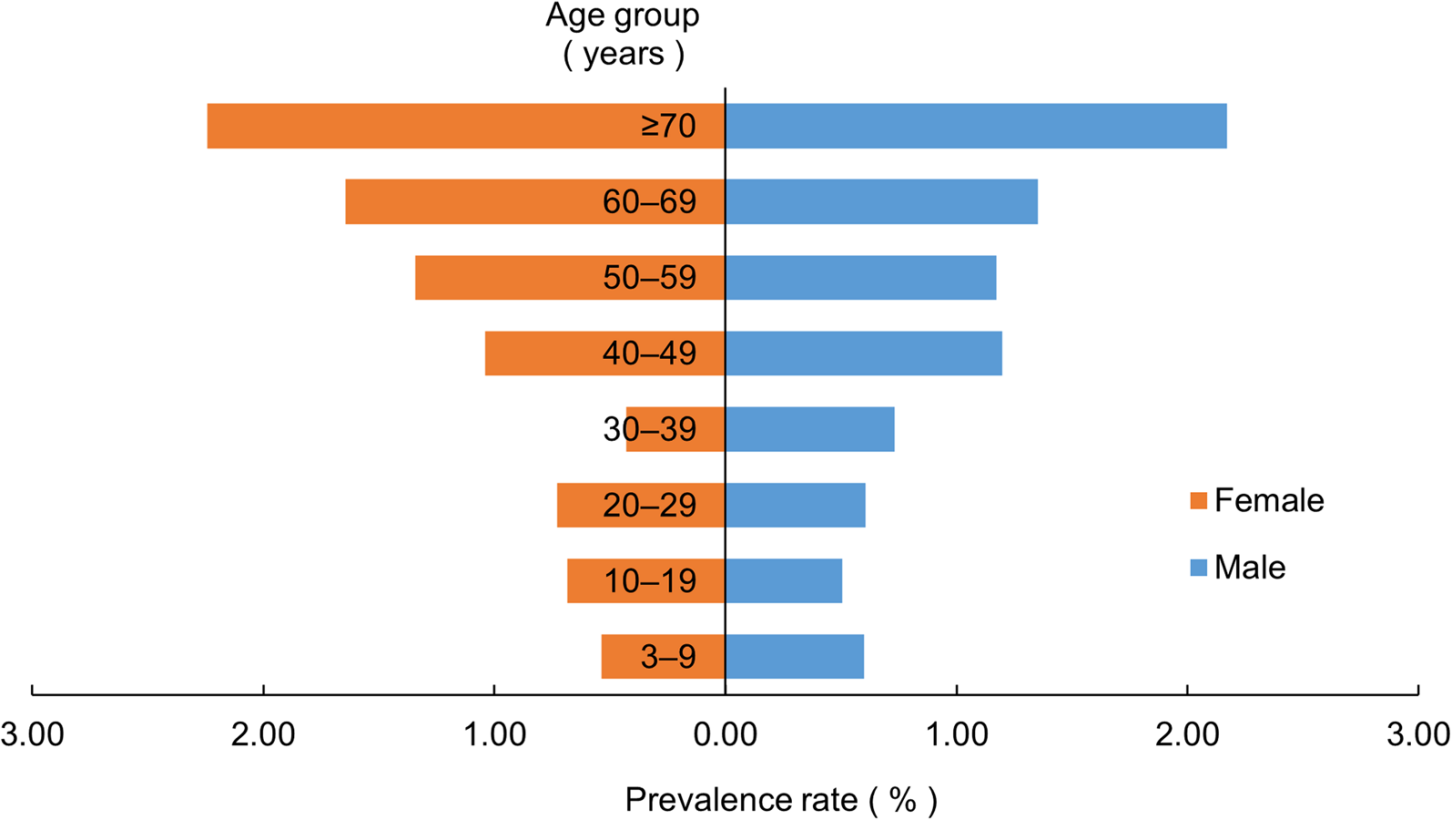
Distribution of STHs prevalence according to sex and age. *STHs* Soil-transmitted helminths

**Fig. 2:**
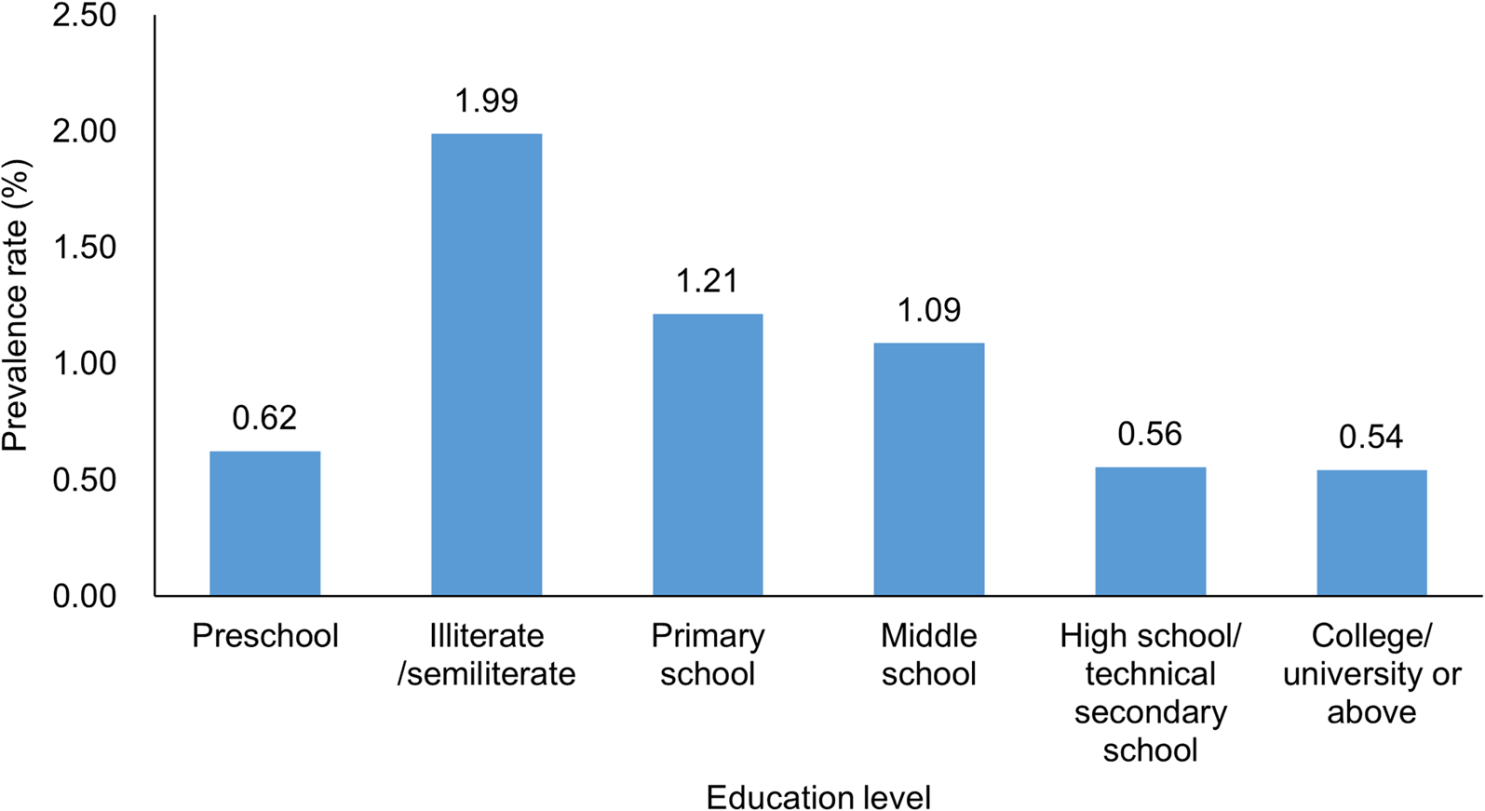
Distribution of STHs prevalence according to educational level. *STHs* Soil-transmitted helminths

The STHs prevalence rate among the male and the female was 1.10% (380/34,525) and 1.17% (439/37,635), respectively, and the difference was not statistically significant (*χ*^2^ = 0.695, *P* = 0.404).The prevalence rate of the ≥ 70-year age group was the highest (2.21%, 198/8968), followed by the 60–69-year age group (1.51%, 196/13,011). The differences among age groups were statistically significant (*χ*^2^ = 187.693, *P* < 0.001), and STHs prevalence rates increased as age increased ($\chi_{{{\text{trend}}}}^{2}$ = 153.499, *P* < 0.001) (Fig. 1). The STHs prevalence rates differed among educational levels (*χ*^2^ = 90.088, *P* < 0.001). The illiterate/semi-illiterate group had the highest prevalence rate (1.99%, 159/7997) and the college/university or above group had the lowest rate (0.54%, 10/1838). STHs prevalence rates decreased as educational level increased ($\chi_{{{\text{trend}}}}^{2}$ = 62.344, *P* < 0.001) (Fig. 2).

**Fig. 3:**
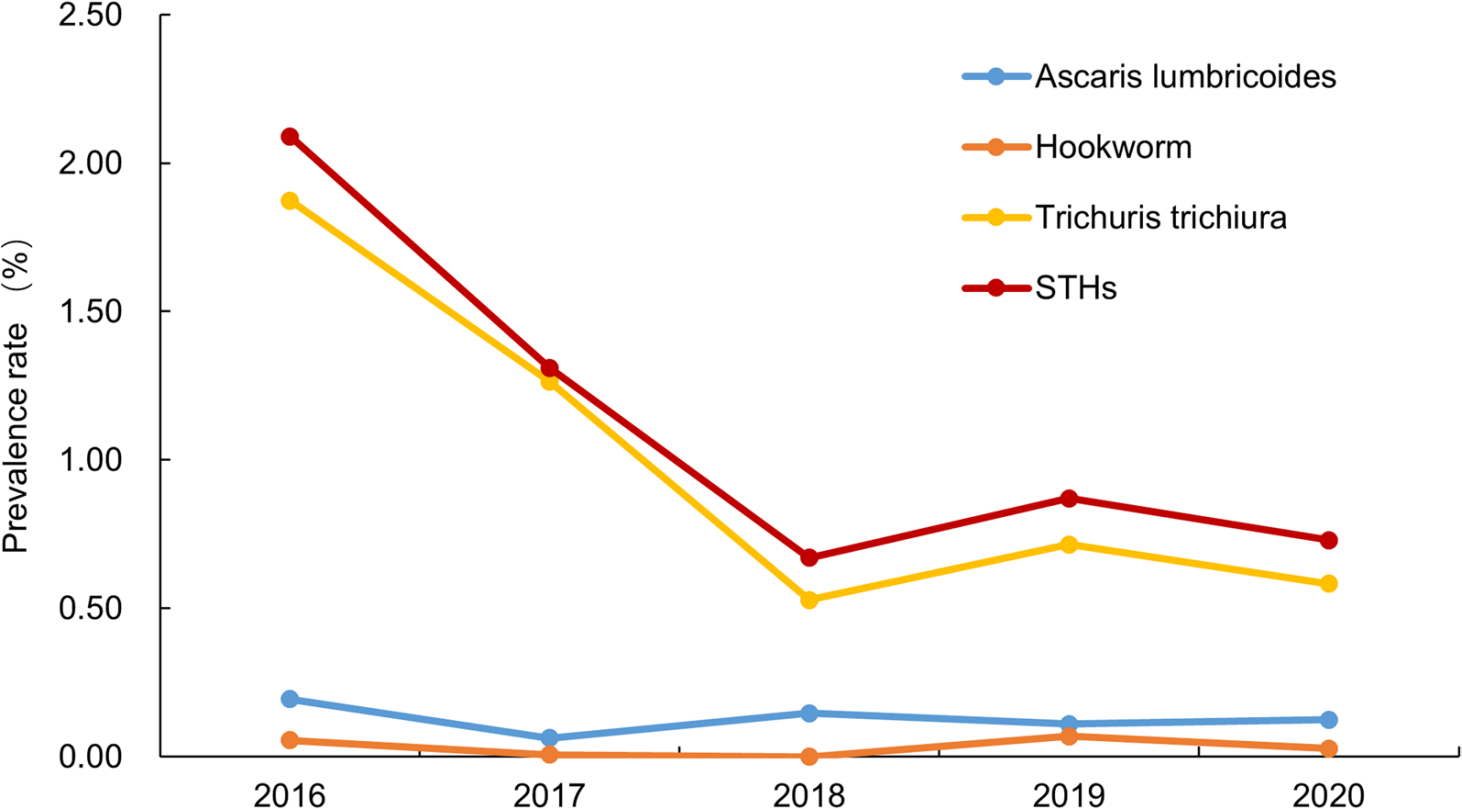
Temporal distribution of STHs prevalence. *STHs* Soil-transmitted helminths

As shown in Fig. 3, the prevalence rates of STHs from 2016 to 2020 were 2.09% (302/14,463), 1.31% (188/14,325), 20.67% (96/14,399), 0.87% (127/14,537) and 0.73% (106/14,436), respectively; the overall trend was linearly decreasing ($\chi_{{{\text{trend}}}}^{2}$ = 127.600, *P* < 0.001), and the difference was statistically significant (*χ*^2^ = 178.749, *P* < 0.001). In terms of species, although the prevalence rates of *A. lumbricoides*, and hookworm were statistically different in different years (*χ*^2^ = 10.404, *P* = 0.034; *χ*^2^ = 16.167, *P* = 0.003), there was no linear trend (all *P* > 0.05). Fortunately, the prevalence rate of *T. trichiura* gradually decreased ($\chi_{{{\text{trend}}}}^{2}$ = 144.323, *P* < 0.001).

**Fig. 4:**
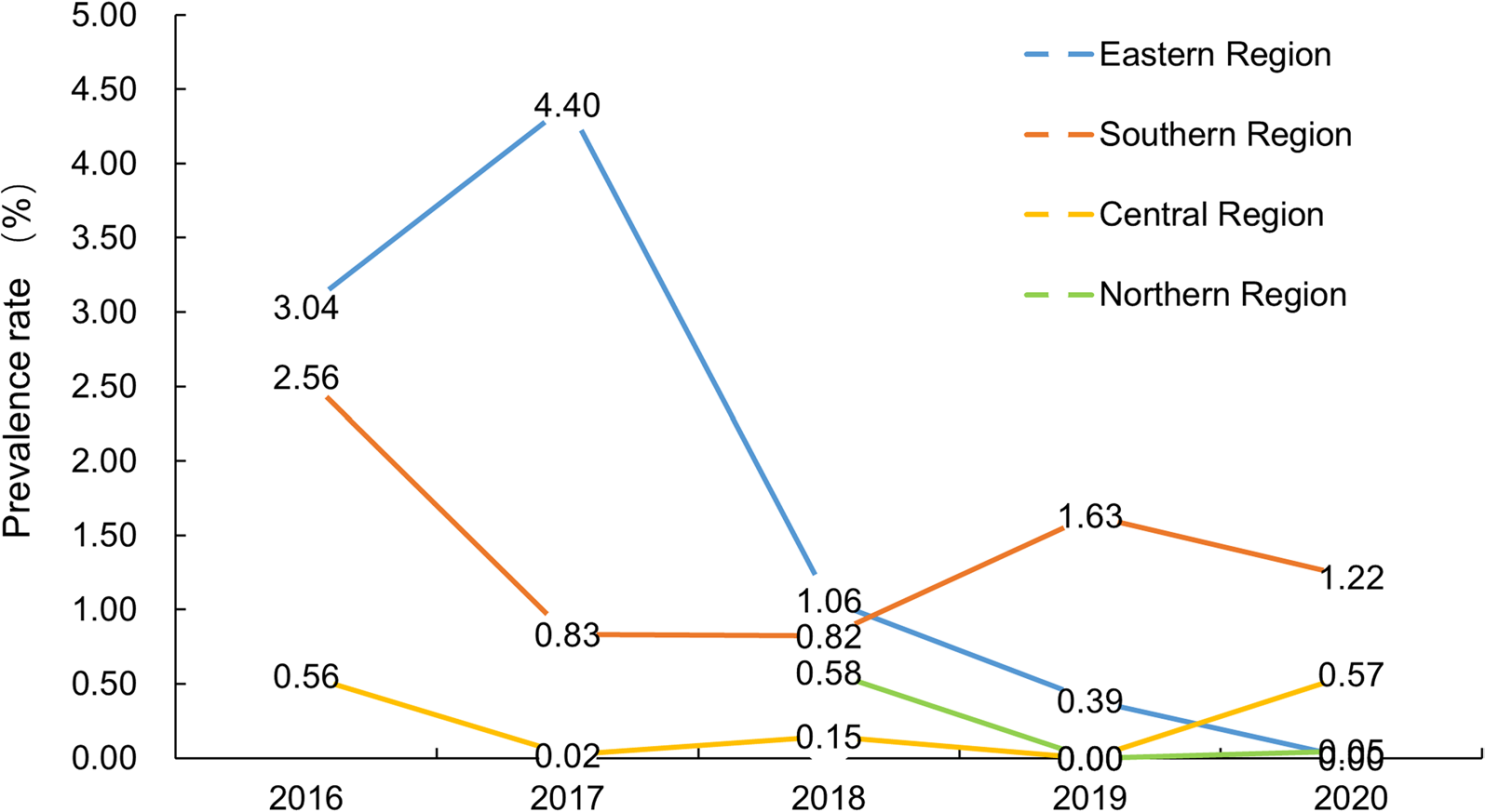
Temporal distribution of STHs prevalence in four regions of Shandong Province

Geographically, the prevalence rate of STHs in the eastern and southern region of Shandong Province showed a linear downward trend ($\chi_{{{\text{trend}}}}^{2}$ = 127.515, 20.218, all *P* < 0.001), while the prevalence rate in the central region did not show a downward trend ($\chi_{{{\text{trend}}}}^{2}$ = 0.023, *P* = 0.880). Notably, one site with a higher STHs prevalence rate (Haiyang > 10%) was included in the surveillance in 2017, and the prevalence rate in eastern region increased accordingly. Similarly, due to the relatively higher prevalence rates of two counties in central region in 2020, the STHs prevalence was higher than in previous years (Fig. 4).

### Knowledge, attitude, and behaviour towards STHs prevention

A total of 1337 participants completed the questionnaire, including 635 males (47.49%) and 702 females (52.51%). The respondents were categorized into three age groups: 255 (19.07%) were in the 10–39 age group, 514 (38.44%) were in the 40–59 age group, and 568 (42.48%) were in the ≥ 60 age group. According to the geographical location, 553 respondents (41.36%) were in northern region, 361 (27.00%) in eastern region, and 423 (31.64%) in southern region of Shandong Province (Additional file 1: Fig. S1).

Five questions regarding prevention knowledge of STHs were investigated. The results showed that 61.78% (826/1337) had heard of STHs, of which 56.30% (465/826) knew the infection routes of roundworm/whipworm, 31.72% (262/826) knew the infection routes of hookworm, 82.20% (679/826) knew the harms of STHs, and 65.62% (542/826) knew the prevention measures for STHs. Through further analysis, the awareness rates of five knowledge in different age groups and different regions were significantly different (all *P* < 0.05), among which the awareness rates of people over 60 years old were the lowest, and the rates of southern region residents were the highest (Table 4).

**Table 4:** Comparison of STHs-related prevention knowledge, attitude, and behaviour in different groups

Group		Awareness rate of STHs-related prevention knowledge (%)					Adoption rate of STHs-related prevention attitudes and behaviours (%)					
		Having heard of STHs	Knowing infection routes of roundworm/whipworm	Knowing infection routes of hookworm	Knowing harms of STHs	Knowing prevention measures for STHs	Washing hands before meals and after defecation	Not consuming raw water	Not fertilizing with fresh stool	Not working on farms barefooted	Willing to take deworming medicine	Willing to change dangerous behaviours
Sex	Male	64.09	56.27	32.92	83.78	65.85	95.91	88.35	84.57	88.82	96.54	54.33
	Female	59.69	56.32	30.55	80.67	65.39	96.01	89.17	85.19	93.16	96.30	59.69
	*χ*^*2*^	2.743	< 0.001	0.538	1.370	0.019	0.010	0.229	0.099	7.758	0.055	3.905
	*P-*value	0.098	0.986	0.463	0.242	0.891	0.922	0.632	0.753	0.005	0.814	0.048
Age Group (year)	10–39	70.98	65.75	41.44	89.50	74.03	98.04	88.24	90.59	92.94	96.08	56.08
	40–59	63.23	57.54	31.38	80.62	65.23	96.11	86.58	88.72	90.66	96.89	60.31
	≥ 60	56.34	49.69	26.56	79.69	61.25	94.89	91.02	78.87	90.67	96.13	54.75
	*χ*^*2*^	16.723	12.453	11.837	8.537	8.409	4.538	5.447	28.354	1.318	0.551	3.549
	*P-*value	< 0.001	0.002	0.003	0.014	0.015	0.103	0.066	< 0.001	0.517	0.759	0.170
Region	Northern	53.71	48.48	26.26	82.49	70.37	97.11	96.20	95.12	96.93	98.55	67.27
	Eastern	64.82	49.15	14.96	77.35	52.99	94.74	83.10	68.98	88.64	96.12	63.99
	Southern	69.74	69.83	50.51	85.76	70.85	95.51	83.92	85.11	85.58	93.85	38.06
	*χ*^*2*^	28.027	34.192	82.525	6.339	23.085	3.492	52.283	116.407	41.737	15.414	92.954
	*P-*value	< 0.001	< 0.001	< 0.001	0.042	< 0.001	0.174	< 0.001	< 0.001	< 0.001	< 0.001	< 0.001

*STHs* soil-transmitted helminths

Among 1337 respondents, 95.96% washed hands before meals and after defecation, 88.78% did not consume raw water, 84.89% did not fertilize with fresh stool, 91.10% did not work on farms barefooted, 96.41% were willing to take deworming medicine, and 57.14% were willing to change dangerous behaviours. This survey showed that, the proportion of not working barefooted on farms (93.16%) and the proportion of willing to change dangerous behaviours (59.69%) in females were higher than that in males (all *P* < 0.05) (Table 4). The proportion of not fertilizing with fresh stool was highest in the 10–39 age group and lowest in the ≥ 60 age group (*P* < 0.05). Except for the behaviour of washing hands before meals and after defecation, the adoption rates of other behaviours and attitudes in the northern region residents were higher than that in eastern and southern regions (all *P* < 0.05).

### Risk factors of STHs infections

Overall, survey on natural and social factors of STHs infection was conducted in 350 villages (Additional file 1: Fig. S1), and the terrains of the villages were predominantly flat (62.57%) or hilly (33.14%). The primary drinking water was tap water (92.29%, 323/350). The total number of toilets was 234,397, of which 155,640 (66.40%) were sanitary toilets, including biogas digesters, three-grid septic tanks, and double-urn funnel toilets, which could harmlessly treat faeces and reduce the spread of STHs.

From 2016 to 2020, the annual average temperatures were (13.83 ± 2.45) °C, (14.09 ± 1.52) °C, (14.79 ± 2.67) °C, (14.18 ± 2.61) °C, and (14.31 ± 1.36) °C in the respective 5 years. The annual average rainfall were (621.75 ± 247.77) mm, (674.70 ± 140.65) mm, (625.10 ± 214.29) mm, (697.29 ± 400.53) mm, and (631.16 ± 248.31) mm, respectively. The proportions of tap water as the primary drinking water were 91.43%, 91.43%, 88.57%, 91.43%, and 98.57%, respectively. The aforementioned differences were not significant (all *P* > 0.05). The proportions of sanitary toilets were 41.91% (15,121/36,081), 47.98% (18,121/37,770), 71.79% (39,115/54,482), 73.20% (31,489/43,020), and 82.16% (51,794/63,044), respectively. The differences were significant (*χ*^2^ = 24,062.161, *P* < 0.001), and the proportions showed linear upward trend ($\chi_{{{\text{trend}}}}^{2}$ = 21,800.313, *P* < 0.001) (Additional file 1: Table S2).

As shown in Table 5, the differences in the annual average temperature, the annual average rainfall, the per capita gross national product (GNP), the annual net income per capita, and the proportion of sanitary toilets were all significant among the four regions of Shandong Province, with southern region demonstrating the highest temperature (14.69 ± 2.14 °C) and rainfall (738.93 ± 299.22 mm) and the lowest GNP (27,027.48 ± 15,258.81 RMB)and annual net income per capita (11,138.97 ± 6463.37 RMB), and eastern region having the greatest proportion of sanitary toilets (81.85%) (all *P* < 0.001).

**Table 5:** Comparison of STHs-related natural and social factors in four regions of Shandong Province

Regions	Annual average temperature (℃, mean ± *SD* )	Annual average rainfall (mm, mean ± *SD*)	Per capita GNP (RMB, mean ± *SD*)	Annual net income per capita (RMB, mean ±* SD*)	Proportion of tap water as main drinking water (%)	Proportion of sanitary toilets (%)
Northern region	13.77 ± 0.96	544.64 ± 195.48	48,194.24 ± 32,656.93	15,432.52 ± 7782.53	96.00 (1/25)	77.30 (9689/12,534)
Eastern region	13.12 ± 1.99	575.28 ± 166.27	79,762.14 ± 50,301.88	22,651.18 ± 18,379.52	86.15 (9/65)	81.85 (52,400/64,023)
Southern region	14.69 ± 2.14	738.93 ± 299.22	27,027.48 ± 15,258.81	11,138.97 ± 6463.37	93.33 (11/165)	59.78 (61,125/102,257)
Central region	14.35 ± 2.43	574.37 ± 220.21	34,166.26 ± 29,033.22	12,121.67 ± 8245.74	93.68 (6/95)	58.34 (32,426/55,583)
*F* / *χ*^*2*^	8.865	13.090	51.216	20.942	4.433	11,144.158
*P-*value	< 0.001	< 0.001	< 0.001	< 0.001	0.218	< 0.001

*STHs* soil-transmitted helminths, *GNP* gross national product, *SD* standard deviation

Classification function coefficients of two infection groups were generated by Bayes discriminant analysis (Table 6). The results showed that terrain was an important factor to both the low and the high infection groups. According to the prediction of the discriminant function, 259 villages (74.00%) in the original group were discriminated correctly, of which 238 villages (74.84%) in the low infection group were discriminated correctly. In the cross-validated group, 256 villages (73.14%) were discriminated into the right group, of which 236 villages (74.21%) in the low infection group were discriminated accurately (Table 7).

**Table 6:** Factors and classification function coefficients of low and high infection group

Groups	Terrain (plain = 1; hills = 2; mountainous area = 3)	Altitude (m)	Annual average temperature (°C)	Annual average rainfall (mm)	Proportion of sanitary toilets (%)	Per capita GNP (¥, RMB)	Annual net income per capita (¥, RMB)	Constant
Low infection group	6.286	− 0.019	0.580	0.120	1.359	0.023	0.029	− 42.788
High infection group	7.268	− 0.021	0.589	0.134	1.213	0.030	0.025	− 46.648

GNP Gross national product

**Table 7:** Classification results of Bayes discriminant analysis

Group		Predicted Group Membership [No. (%)]		Total
		Low infection group	High infection group	
Original	Low infection group	238 (74.84)	80 (25.16)	318 (100.00)
	High infection group	11 (34.38)	21 (65.63)	32 (100.00)
Cross-validated^a^	Low infection group	236 (74.21)	82 (25.79)	318 (100.00)
	High infection group	12 (37.50)	20 (62.50)	32 (100.00)

74.00% of original grouped cases correctly classified

73.14% of cross-validated grouped cases correctly classified

^a^Cross validation is done only for those cases in the analysis. In cross validation, each case is classified by the functions derived from all cases other than that case

## Discussion

In 2016, the “National Plan for Prevention and Control of Echinococcosis and other Key Parasitic Diseases (2016–2020)” was launched with the aim to establish and improve the surveillance system for key parasitic diseases and reduce the parasite prevalence rate by the end of 2020. Since 2016, Shandong Province has expanded the surveillance scope and 14 counties were included in STHs surveillance every year. From 2016 to 2020, the STHs prevalence rates had shown a downward trend and had dropped to a low level, achieving the established goals.

Shandong Province has taken a series of prevention and control measures to reduce the prevalence of STHs. Publicity materials that both professional and interesting were distributed under the requirements of target populations [30]. Meanwhile, new media were wildly used to promote the population’s awareness level of STHs-related prevention knowledge and to improve their self-protection abilities [31, 32]. Additionally, professionals visited primary schools annually and conducted themed education sessions on parasitic disease prevention to improve students and their families’ awareness [33, 34]. From 2016 to 2020, nearly 800,000 people had received health education in Shandong Province. Provincial-level competitions were organized to promote practicing of parasitic disease prevention and control skills. Similarly, county-level and city-level competitions were conducted [35, 36]. The training coverage was expanded through level-by-level competitions, and the professional teams were strengthened [37]. Over 46,000 professionals had received training on parasitic diseases prevention and control skills from 2016 to 2020. Combined with the “Patriotic Health Movement”, Shandong Province had carried out a series of activities to promote rural toilet reform, to strengthen rural drinking water safety. This study showed that the coverage rate of sanitary toilets increased from 41.91% in 2016 to 82.16% in 2020, and the coverage rate of tap water increased from 91.43% in 2016 to 98.57% in 2020. Thus, the quality of rural environmental sanitation improved significantly, which greatly reduced the transmission of STHs [38].

In this study, we found *T. trichiura* was the predominant infection species of STHs infection in Shandong Province. The 1988–1992 national survey showed that *A. lumbricoides* infection was predominant in Shandong Province, and the prevalence rate of *A. lumbricoides* was highest in the 5 to 14-year age group (42.8% to 52.2%) [26]. To reduce *A. lumbricoides* prevalence, Shandong Province administered mass deworming with albendazole in primary and middle school students at 200 mg/person and 400 mg/person, respectively, from 1996 to 2000 [39].Since the national survey from 2001 to 2004, the primary infection species in Shandong Province has changed to *T. trichiura*, owning to the greatly reduced prevalence rate of *A. lumbricoides* [27, 40–43]. Related researches found that, *A. lumbricoides* and *T. trichiura* have different sensitivities to common anthelmintics, and the WHO-recommended drugs for the treatment of STHs infection, e.g. albendazole, mebendazole, levamisole, and pyrantel pamoate [44, 45], did not show acceptable efficacies against *T. trichiura* at single doses [46–48]. However, all of them showed acceptable efficacies against *A. lumbricoides* [49]. This may be one of the reasons why the decline rate of *T. trichiura* prevalence was lower than that of *A. lumbricoides*. Therefore, it is necessary to strengthen the development of efficient and safe new drugs for *T. trichiura* eradication [47, 48, 50].

Worthy of note is the unbalanced regional distribution of STHs infection. The prevalence rate was higher in eastern and southern region than in other regions of Shandong Province, which was consistent with the previous three national surveys [26, 27, 43]. Spatial scan analysis revealed 4 clusters, three located in eastern region and one in southern region. The prevalence of STHs is known to be related to natural factors, such as temperature, humidity, and geographical environment [51–53]. STHs prevalence is also closely related to social factors, such as local economic level, health status, living habits, production mode, and health-related self-awareness [54–59]. The third national survey from 2014 to 2016 showed that the population STHs prevalence rate decreased as the economic level increased [60]. This study found that the economic level of southern region was lower than other regions and the popularization rate of sanitary toilets in southern region was low, potentially explaining the high STHs prevalence rate in this region. Therefore, the construction of sanitary toilets and their standardized use should be strengthened in southern region. However, the economic income of residents in eastern region was the highest, and the proportion of sanitary toilets was the largest in Shandong Province, but the STHs prevalence rate in this region remained high. Further investigations revealed that the awareness rate of knowledge and the adoption rate of attitudes and behaviours towards STHs prevention were all low in eastern region. Therefore, health education on prevention of STHs infection should be strengthened in eastern region [61, 62].

This study showed that STHs prevalence rate increased with age, and the highest rate was found in people over 70 years, followed by those over 60 years, consisting with recent national surveillance [20, 63–65]. And this study also found that the awareness rate of STHs-related prevention knowledge in population aged ≥ 60 was lower than population aged < 60, and the proportion of using fresh manure fertilization in the ≥ 60 age group was much higher than population aged < 60, indicating the old was a potential risk factor for STHs prevalence. Health education should be strengthened on considering the characteristics and acceptance of the elder population, to help them change their dangerous production and living habits. [66, 67]. Currently, in rural areas, people aged ≥ 60 predominantly engage in agricultural production [68], and their habits are difficult to change through single health education [69]. Therefore, comprehensive prevention and control measures of drug deworming and environmental improvement are required to reduce STHs prevalence in the elder age group.

In this study, we obtained five consecutive years of surveillance data from widely distributed surveillance sites and a large sample size. We also conducted comparisons with the three previous national surveys and systematically analyzed the STHs prevalence characteristics and trends in Shandong Province. We implemented questionnaire survey on STHs-related knowledge, attitude, and behaviour and related natural and social factors to explore the factors influencing STHs prevalence in Shandong Province profoundly. The aspects as mentioned above represent the advantages and strengths of this study.

Nevertheless, this study has some limitations. Only natural and social factors were included in the discriminant analysis, and the prediction accuracy of the discriminant function required further improvement.

## Conclusions

This study analyzed the STHs prevalence trend and risk in Shandong Province, China. The results showed that STHs prevalence rate has dropped to a low level but it is worth noting that *T. trichiura* is still prevalent in some parts of Shandong Province. Affected by social-economic factors and people’ cognitive and behavioural factors, respectively, STHs prevalence rates in southern and eastern regions were still high. And the elderly were susceptible to be infected with STHs owning to their low awareness level of knowledge towards STHs prevention and high adoption rate of dangerous production and living behaviours. Therefore, integrated approaches of health education, environment improvement and behaviour change should be strengthened to obtain a persistently downward trend of STHs prevalence and finally achieve the STHs transmission interruption in China.

## Supplementary Information

**Additional file 1: Figure S1.** Total results of STHs surveillance and questionnaire survey in Shandong Province. **Table S1.** The two-pair comparison of STHs prevalence between different regions of Shandong Province. **Table S2.** Comparison of STHs-related natural and social factors in different years

## Data Availability

Not applicable.

## Abbreviations

STHs: Soil-transmitted helminthes
CDC: Center for Disease Control and Prevention
GNP: Gross national product
*LLR*: Log-likelihood ratio
*RR*: Relative risk
WHO: World Health Organization
