# Supplementary material for: The prevalence of soil transmitted helminths and its influential factors in Shandong Province, China: an analysis of surveillance data from 2016 to 2020
Source: Infect Dis Poverty. 2023 May 22;12:54. doi: 10.1186/s40249-023-01100-4 (PMC10201724; doi:10.1186/s40249-023-01100-4)
Supplement: Supplementary file 1 — Additional file 1: Figure S1. Total results of STHs surveillance and questionnaire survey in Shandong Province. Table S1. The two-pair comparison of STHs prevalence between different regions of Shandong Province. Table S2. Comparison of STHs-related natural and social factors in different years [file 40249_2023_1100_MOESM1_ESM.docx]

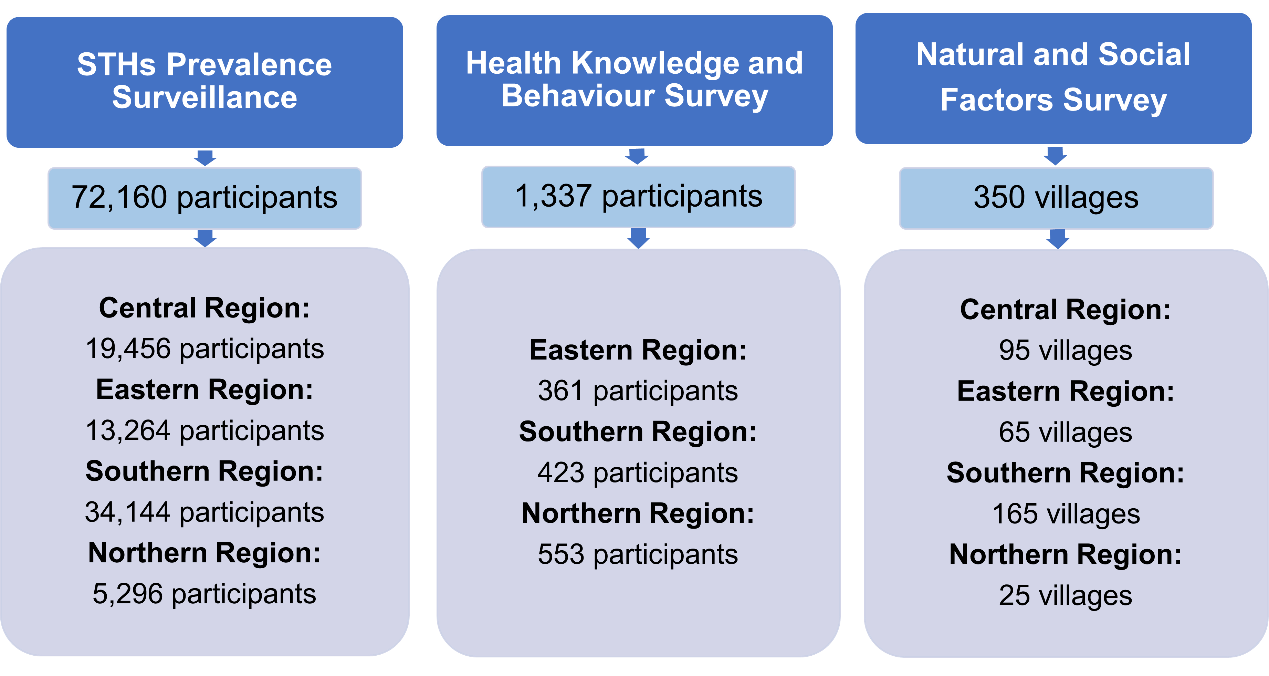


**Figure S1** Total results of STHs surveillance and questionnaire survey in Shandong Province

**Table S1** The two-pair comparison of STHs prevalence between different regions of Shandong Province

| **Region** | **Prevalence rate**  **(%)** | **Statistical indicators** | **Central** | **Eastern** | **Southern** | **Northern** |
| --- | --- | --- | --- | --- | --- | --- |
| Central | 0.25 | *χ^2^* | 0 | 259.456 | 179.418 | 2.463 |
|  |  | *P* | 1.000 | < 0.001 | < 0.001 | 0.117 |
| Eastern | 2.02 | *χ^2^* | 259.456 | 0 | 19.427 | 92.455 |
|  |  | *P* | < 0.001 | 1.000 | < 0.001 | < 0.001 |
| Southern | 1.45 | *χ^2^* | 179.418 | 19.427 | 0 | 63.495 |
|  |  | *P* | < 0.001 | < 0.001 | 1.000 | < 0.001 |
| Northern | 0.13 | *χ^2^* | 2.463 | 92.455 | 63.495 | 0 |
|  |  | *P* | 0.117 | < 0.001 | < 0.001 | 1.000 |

*P*-values were adjusted for multiple comparisons using the Bonferroni correction

**Table S2** Comparison of STHs-related natural and social factors in different years

| **Year** | **Annual average temperature (x±S, ℃)** | **Annual average rainfall (x±S, mm)** | **Proportion of tap water as main drinking water  (%)** | **Proportion of sanitary toilets  (%)** |
| --- | --- | --- | --- | --- |
| 2016 | 13.83 ± 2.45 | 621.75 ± 247.77 | 91.43 | 41.91  (15 121/36 081) |
| 2017 | 14.09 ± 1.52 | 674.70 ± 140.65 | 91.43 | 47.98  (18,121/37,770) |
| 2018 | 14.79 ± 2.67 | 625.10 ± 214.29 | 88.57 | 71.79 (39,115/54,482) |
| 2019 | 14.18 ± 2.61 | 697.29 ± 400.53 | 91.43 | 73.20  (31,489/43,020) |
| 2020 | 14.31 ± 1.36 | 631.16 ± 248.31 | 98.57 | 82.16 (51,794/63,044) |
| *F* / *χ^2^* | 1.836 | 1.158 | 5.458 | 24,062.16 |
| *P-*value | 0.121 | 0.329 | 0.243 | <0.001 |

STHs Soil-transmitted helminths
